# Predictors of large cell transformation in patients with Sezary Syndrome—A retrospective analysis

**DOI:** 10.1371/journal.pone.0277655

**Published:** 2022-11-16

**Authors:** Neil K. Jairath, Redina Bardhi, John S. Runge, Ramona Bledea, Ruple Jairath, Yang Wang, Matthew Patrick, Ryan A. Wilcox, Alexandra C. Hristov, Lam C. Tsoi, Trilokraj Tejasvi

**Affiliations:** 1 Department of Dermatology, University of Michigan, Ann Arbor, MI, United States of America; 2 Department of Dermatology, Wayne State University, Detroit, MI, United States of America; 3 Department of Dermatology and Venereology, Peking University First Hospital, Beijing, China; 4 Department of Hematology and Oncology, University of Michigan, Ann Arbor, MI, United States of America; 5 Department of Pathology, University of Michigan, Ann Arbor, MI, United States of America; 6 Department of Computational Medicine and Bioinformatics, Department of Biostatistics, University of Michigan, Ann Arbor, MI, United States of America; 7 Veteran Affairs Health System, Ann Arbor, MI, United States of America; Postgraduate Institute of Medical Education and Research, INDIA

## Abstract

**Background:**

Large cell transformation (LCT) of Sezary Syndrome (SS) is a rare phenomenon. To date, there are no rigorous studies identifying risk factors for its development.

**Objectives:**

Here, we seek to characterize the clinicopathologic risk factors that predispose patients with SS to develop LCT.

**Methods:**

We retrospectively evaluated all SS patient records available in the Michigan Medicine Cancer Registry from 2010–2021. Clinical and pathologic variables were compared between groups. The Kaplan-Meier method and log-rank test were used to assess overall survival.

**Results:**

Of 28 SS patients identified, eight patients experienced LCT, and 20 did not (NLCT). Peak lactate dehydrogenase (LDH) before LCT (p = 0.0012), maximum total body surface area (TBSA) involvement before LCT (p = 0.0114), absolute CD8+ cell count measured on flow cytometry at diagnosis of SS (p = 0.0455) and at the most recent blood draw (p = 0.00736), and ulceration on biopsy (p = 0.0034) were significant clinicopathologic variables identified between the SS patients that developed LCT versus those that did not.

**Conclusions:**

Maximum TBSA involvement, peak LDH, presence of ulceration, and decreased levels of CD8+ cells in the peripheral blood may predict the development of LCT in patients with SS.

## Introduction

Sezary syndrome (SS), commonly referred to as the leukemic variant of mycosis fungoides (MF), constitutes fewer than 5% of cutaneous T cell lymphoma CTCL [1]. The WHO/EORTC classifies SS as disease process characterized pathologically by the presence of Sezary cells (neoplastic T‐cells) in any or all of the skin, lymph nodes, and peripheral blood, and on physical examination by generalized lymphadenopathy, erythroderma [2]. SS is associated with very poor survival and a variable disease course, which may involve rapid progression in a subset of patients, with 5-year survival approximated at 11% [3,4].

“Large Cell Transformation” (LCT) can be diagnosed by the presence of large cells (defined as lymphocytes ≥ 4 times the size of normal lymphocytes) in ≥ 25% of the dermal infiltrate, or a specimen that demonstrates large cell nodules [5,6]. LCT can take place in up to 20% of advanced MF cases, and generally correlates with a more aggressive disease course [7]. Along with CTCL, patients with SS may also undergo LCT, and the median survival time for large cell transformed-SS ranges from 19–36 months [8,9]. Unfortunately, our current understanding of the molecular mechanisms and clinicopathologic features that correlate with progression to LCT is limited [7].

Due to the low incidence of this disease, and the poor prognosis associated with its transformation, there is a stark paucity of data regarding risk factors for the progression of SS to LCT. For this reason, there is a distinct need to better understand the clinicopathologic risk factors that might signal SS transformation to LCT prior to diagnosis. This knowledge is an essential first step to developing targeted management and therapies for these patients, and preventing the complications associated with more aggressive disease courses. Here, we seek to retrospectively characterize the clinicopathologic risk factors that may predispose patients with SS to LCT. We present the following article/case in accordance with the STROBE reporting checklist.

## Materials and methods

This study was conducted with the approval of the Institutional Review Board at the University of Michigan. The need for informed consent was waived in this study and all data were anonymized prior to access. We retrospectively evaluated all SS patient records available at the Michigan Medicine from 2010–2021. The patients were queried from the University of Michigan electronic health record database and identified using free text parameters that included any of the words ‘progression’, or ‘large cell’ associated with a diagnosis of ‘Sezary Syndrome’, or ‘cutaneous T-cell lymphoma’ (‘CTCL’). Only patients with at least one histopathologically confirmed biopsy specimen for each diagnosis, reviewed by a dermatopathologist/hematopathologist with cutaneous lymphoma expertise to confirm the findings (A.H.), were included in the study. Cases which met the above criteria were then clinically confirmed as SS by N.K.J., R.B., and T.T. via detailed electronic health record review, which included immunohistochemical and molecular data alongside the consensus of cutaneous lymphoma experts in dermatology and hematology (T.T. and R.A.W.).

All patients were classified according to the International Society for Cutaneous Lymphomas (ISCL) and European Organization of Research and Treatment of Cancer (EORTC) revised criteria of 2007 [6]. Disease staging was determined by documented physical examination, peripheral blood smear, flow cytometry, complete blood count and chemistries, and imaging that included computed tomography (CT) and/or positron emission tomography (PET) scan of the chest, abdomen, and pelvis.

### Clinical and pathological data

Patient records, documented imaging, available histopathology slides and reports, as well as flow cytometric and molecular data were all reviewed. Clinicopathologic factors hypothesized to correlate with progression to LCT which were collected for analysis included: age and date at diagnosis of both SS and LCT, sex, race, ethnicity, clinical stage at diagnosis and subsequent visits, clinical findings at first diagnosis, first histopathologic documentation of SS and LCT, dates of death or last follow up, location/number of biopsy sites, maximum total body surface area (TBSA) involvement before LCT, presence of clinically or radiologically-apparent lymphadenopathy, peak serum lactate dehydrogenase prior to and at diagnosis of LCT, complete blood count at diagnosis of Sezary Syndrome and LCT, evidence of clonal T-cell rearrangements in both tissue and blood samples, medication and treatment history specific to SS and LCT, as well as treatment duration and response. Response to treatment was classified as either complete remission, partial response, stable disease, or progressive disease, assessed clinically according to the ISCL/EORTC guidelines6.

Based on record review of histopathology reports, the following histopathologic features were recorded and analyzed: locations/dates of all recorded biopsies, presence of LCT, presence of neoplastic T-cells (Sezary cells), presence of folliculotropism, fibrosis, ulceration, epidermotropism, Pautrier microabscesses, spongiosis, neutrophil infiltration, and follicular mucin. Expression of CD2, CD5, CD7, CD30, and the CD4:CD8 ratio were also evaluated via immunohistochemistry in the primary tumor samples. In addition, flow cytometry of the peripheral blood at diagnosis of SS and LCT, absolute and relative values for CD1a, CD2, CD3, CD4, CD4+CD7-, CD4+CD26-, CD4+CD7-CD26-, CD5, CD7, CD8, CD19, CD20, NK Cells, and the CD4:CD8 ratio, were all included for analysis.

Collected peripheral blood samples were stained with the 10-color combination CD1a-V500c/CD2-PE/CD3-BV605/CD4–V450/CD5-PERCPCy5.5/CD7–APC/CD8-FITC/CD19-PECy7/CD20-APCH7/CD26-APCR700 and lysed with FACSlyse following the manufacturer’s directions (Becton Dickinson [BD], San Jose, CA). After washing, cells were analyzed on a FACSCalibur flow cytometer (BD). Photomultiplier tube voltages were established using QC3 beads (Bangs Laboratories, Fishers, IN) per the manufacturer’s instructions. Compensation was set manually before acquisition, and voltages and compensation were validated using a known normal sample. A minimum of 10,000 ungated events was acquired, but in all cases, an acquisition gate was set on displays of CD3 vs side scatter to acquire at least 2,500 CD3+ T cells.

### Statistical analysis

Analyses were executed using software packages from the R Foundation for Statistical Computing (Vienna, Austria). The Mann-Whitney U test and Fisher’s Exact Test were used to compare variables between different groups. The Kaplan-Meier method was used to assess overall survival (OS) and time to next treatment (TTNT) and differences were compared using the Mantel-Cox log rank test. OS was defined as the time from SS diagnosis to death. TTNT was defined as mean time to next systemic treatment. For continuous variables analyzed using the Kaplan-Meier method, the median value for all patients in the dataset was calculated, and designations of “High” or “Low” were given based on whether the value for each individual patients was higher or lower than the sample median, respectively.

## Results

### Clinical features

**Fig 1:**
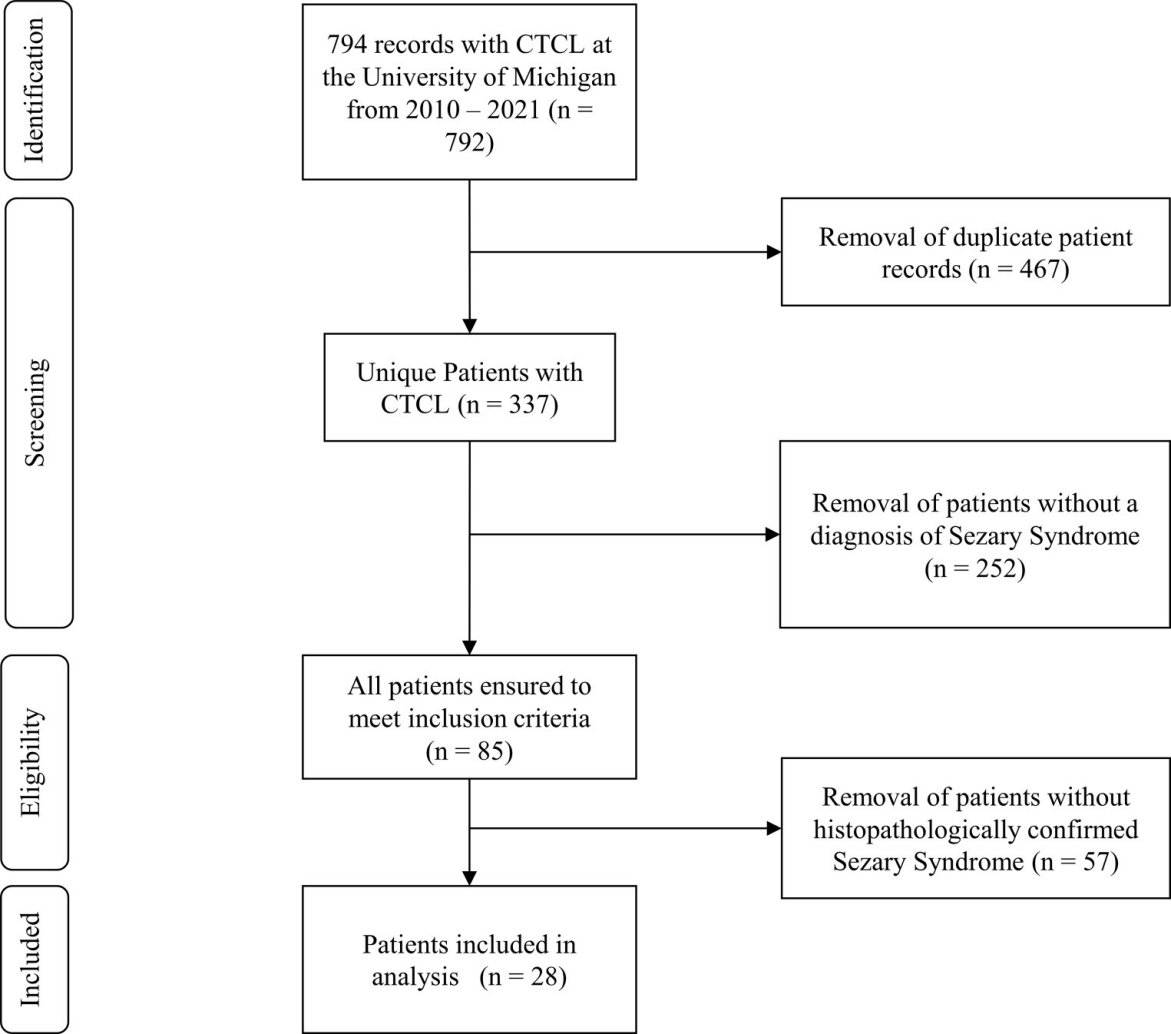
Flow diagram detailing inclusion and exclusion of patients at each stage of the collection.

Medical records from 794 entries in 337 unique patients were initially retrieved using the aforementioned search strategy. Of these, 85 patients were found to have either SS or LCT diagnosis. The authors manually reviewed these 85 patient records and excluded any patients that did not have a retrievable confirmed histopathologic or hematological diagnosis of SS. After applying exclusion criteria, 28 patients were included in the final analysis (**Fig 1**). The median age at diagnosis of SS was 72 (range 46–99). The diagnosis of SS preceded the diagnosis of LCT in all cases in which transformation occurred. At the time of data analysis, 18 of the 28 (64%) patients in the study had died, and 10 (36%) were alive. Additional malignant diagnoses were present in 2 patients. One patient (NLCT group, deceased secondary to lung adenocarcinoma) had a history of metastatic lung adenocarcinoma, concurrent with their SS diagnosis, and one patient had a history of metastatic melanoma concurrent with their SS diagnosis and preceding chronic lymphocytic leukemia (POT-1 mutation, LCT group, deceased secondary to metastatic melanoma). Concise demographic tables and summary statistics can be visualized in **Table 1A.**

**Table 1:** A. Concise demographic details for each patient included in the study. “NA” in the “Days from SS Dx to death” column indicates that the patient was still alive at last follow up. B. Concise flow cytometric and clinicopathologic characteristics for each patient included in the study.

NLCT	Status	Days from SS Dx to death	Sex	Race	Stage at dx	Age at SS dx
Patient 1**	Dead	69	Female	Caucasian	1B	63
Patient 2	Dead	847	Female	Caucasian	1B	72
Patient 3**	Dead	396	Male	Caucasian	3A	72
Patient 4	Dead	2275	Male	Caucasian	3B	84
Patient 5	Dead	1558	Female	Caucasian	3B	88
Patient 6	Dead	158	Female	African American	4A2	91
Patient 7**	Alive	NA	Male	Caucasian	3B	58
Patient 8**	Alive	NA	Male	Caucasian	1B	70
Patient 9	Alive	NA	Female	Caucasian	4A2	73
Patient 10**	Alive	NA	Female	Caucasian	3	74
Patient 11	Alive	NA	Male	Caucasian	3	76
Patient 12	Dead	338	Female	Caucasian	3	76
Patient 13**	Dead	997	Female	Caucasian	3A	81
Patient 14**	Dead	239	Male	Caucasian	3A	74
Patient 15**	Alive	NA	Male	Caucasian	3B	65
Patient 16**	Alive	NA	Female	African American	3A	99
Patient 17**	Alive	NA	Male	Caucasian	1B	60
Patient 18**	Dead	326	Female	Caucasian	3A	56
Patient 19**	Dead	127	Male	Caucasian	1B	74
Patient 20**	Alive	NA	Female	Caucasian	3A	71
LCT	Status	Days from SS Dx to death	Sex	Race	Stage at dx	Age at SS dx
Patient 21**	Dead	419	Male	Caucasian	1B	71
Patient 22	Alive	NA	Male	Caucasian	3	74
Patient 23	Dead	952	Male	Caucasian	3	48
Patient 24**	Dead	1365	Female	Caucasian	3B	88
Patient 25**	Dead	550	Male	Caucasian	2B	59
Patient 26	Dead	1631	Male	Caucasian	3	74
Patient 27**	Dead	736	Female	African American	2B	46
Patient 28	Dead	444	Female	Caucasian	4A2	47
Summary Statistics	Status	Days from SS Dx to death	Sex	Race	Stage at dx	Age at SS dx
*Notes	Dead/Alive		Male/ Female	Caucasian/African-American	Stage 1 or 2/Stage 3/Stage 4	
NLCT Number	11/9*		9/11*	18/2*	5/13/2*	
LCT Number	7/1*		5/3*	7/1*	3/4/1*	
NLCT Avg		666.4				74
LCT Avg		871				63
NLCT Max		2275				99
LCT Max		1631				88
NLCT Min		69				56
LCT Min		419				46
NLCT Median		338				73
LCT Median		736				65

NLCT	CD8, Abs (Most Recent)	CD8, Abs (at SS dx)	CD8, Relative Fraction (at LCT dx)	Max TBSA (%)	Ulceration	Peak LDH	CD4:CD8 Ratio in Primary Tumor
Patient 1	236	236	NA	35	No	302	>10:1
Patient 2	345	216	NA	45	No	279	5–7:1
Patient 3	152	533	NA	50	No	698	NA
Patient 4	195	832	NA	65	No	288	10:1
Patient 5	216	187	NA	25	No	259	5–10:1
Patient 6	643	389	NA	55	No	440	>10:1
Patient 7	47	253	NA	40	No	521	>10:1
Patient 8	1642	1642	NA	35	No	226	1–2:1
Patient 9	521	1239	NA	80	No	635	>10:1
Patient 10	38	126	NA	90	No	259	4–5:1
Patient 11	25	25	NA	50	No	274	>10:1
Patient 12	556	556	NA	60	No	500	NA
Patient 13	220	313	NA	80	No	292	NA
Patient 14	1505	1432	NA	10	No	541	8–10:1
Patient 15	152	173	NA	40	No	212	4–5:1
Patient 16	122	122	NA	50	No	446	>10:1
Patient 17	526	2731	NA	50	No	279	1:1–2
Patient 18	674	657	NA	65	No	681	>10:1
Patient 19	1445	1445	NA	35	No	289	5–7:1
Patient 20	262	262	NA	40	No	302	NA
LCT	CD8, Abs (Most Recent)	CD8, Abs (at SS dx)	CD8, Relative Fraction (at LCT dx)	Max TBSA (%)	Ulceration	Peak LDH	CD4:CD8 Ratio in Primary Tumor
Patient 21	93	113	5	85	No	2419	7–10:1
Patient 22	112	112	4	80	No	567	7:1
Patient 23	67	156	4	100	No	963	>10:1
Patient 24	24	70	7	100	Yes	587	<10:1
Patient 25	NA	NA	NA	90	Yes	683	>10:1
Patient 26	42	NA	4	80	Yes	581	>10:1
Patient 27	24	99	1	10	No	1121	5:1
Patient 28	58	58	7	70	Yes	553	<10:1
Summary Statistics	CD8, Abs (Most Recent)	CD8, Abs (at SS dx)	CD8, Relative Fraction (at LCT dx)	Max TBSA (%)	Ulceration	Peak LDH	CD4:CD8 Ratio in Primary Tumor
*Notes			Recent NLCT vs. LCT at dx				Greater than 10:1
NLCT Number					0		7*
LCT Number					4		3*
NLCT Avg	476	668	N/A	50		386	
LCT Avg	60	101	5	77		934	
NLCT Max	1642	2731	N/A	90		698	
LCT Max	112	156	7	100		2419	
NLCT Min	25	25	N/A	10		212	
LCT Min	24	58	1	10		553	
NLCT Median	249	351	N/A	50		297	
LCT Median	58	106	4	83		635	
U Test p-value	**0.00736** **	**0.0455** **	**0.0385** **	**0.0114** **		**0.0012** **	
Fisher’s Exact Test	** **				**0.0034** **		

Abbreviations: LCT, Large Cell Transformation; NLCT, Non-Large Cell Transformation; SS, Sezary Syndrome; Dx, diagnosis; Max, Maximum; Min, Minimum.

Abbreviations: LCT, Large Cell Transformation; NLCT, Non-Large Cell Transformation; SS, Sezary Syndrome; Abs, Absolute; TBSA, Total Body Surface Area; LDH, Lactate Dehydrogenase; Dx, diagnosis; Max, Maximum; Min, Minimum.

** Denotes significance, *p* < 0.05.

### Pathological features

All patients included in the study were required to have had at least one histopathologically-confirmed biopsy compatible with a diagnosis of SS (along with flow-cytometric analysis and/or TCR rearrangement). All patients diagnosed with LCT were required to have had at least one histopathologically-confirmed biopsy demonstrating evidence of LCT. Eight patients had biopsies showing LCT following or concurrent with a confirmed SS diagnosis, while 20 patients had only a histopathologically-confirmed diagnosis of SS, non-transformed. Certain features, such as folliculotropism, epidermotropism, fibrosis, ulceration, Pautrier microabscesses, spongiosis, neutrophils, and follicular mucin were recorded if they were present on any biopsy specimens.

### Associations of clinicopathologic variables with development of LCT

Clinical variables in patients who underwent transformation that showed statistically significant differences between the two groups (transformed and non-transformed) included mean peak LDH value before LCT (934.3 vs. 386.2 U/L, p = 0.0012, reference range 105–333 U/L), and mean maximum TBSA involvement before diagnosis of LCT, measured either via the Palmer Method or Wallace Rule of Nines (76.9% vs. 50.0%, p = 0.0114). Given the retrospective nature of the analysis, standardized TBSA methodology was not feasible. Absolute CD8+ cell count in LCT vs. non-LCT groups at diagnosis of Sezary Syndrome and prior to LCT (101 vs. 668 cells/cmm, p = 0.046) was significantly different between the groups. When comparing flow cytometry results at the diagnosis of LCT syndrome (for LCT patients) against the most recent blood draw for patients who had not developed LCT by the end of data collection, there was a significant difference in the mean percentage of CD8+ cells (4.573% vs. 14.12%, p = 0.0385). Due to the variability of time of LCT, it was also decided that data should be analyzed “at most recent collection” to allow for as standardized an approach to follow-up analysis as possible. At most recent collection, mean absolute CD8+ cell count in the LCT versus non-LCT group (60 vs. 476 cells/cmm, p = 0.0074) was significantly different between the two groups. An ROC analysis deemed a cutoff of 120 CD8+ cells/mm to be an appropriate stratification point. At time of SS diagnosis, 71.4% (5/7) of patients with absolute CD8 count less than 120 cells/mm went on to develop LCT, whereas 94.7% (18/19) patients with cell counts above 120 cells/mm did not develop LCT. At most recent blood draw, 70% (7/10) patients with absolute CD8 cell count less than 120 cells/cmm had developed LCT, whereas 100% (17/17) of patients with cell counts above 120 cells/mm did not develop LCT.

Pathological variables in patients who underwent transformation that showed statistically significant differences between the two groups included presence of ulceration on any specimens prior to development of LCT (p = 0.0034), with greater incidence of ulceration in patients with LCT. Two patients with ulceration on pathologic specimen were also noted to have clinically ulcerated lesions. Patients with ulceration also had greater average maximum TBSA involvement, (LCT = 53.1% vs. NLCT = 85%, p = 0.008). Results of the significant differences in clinicopathologic variables between the two groups and summary statistics can be visualized in **Table 1B.**

### Survival and time-to-next-treatment analysis

**Fig 2:**
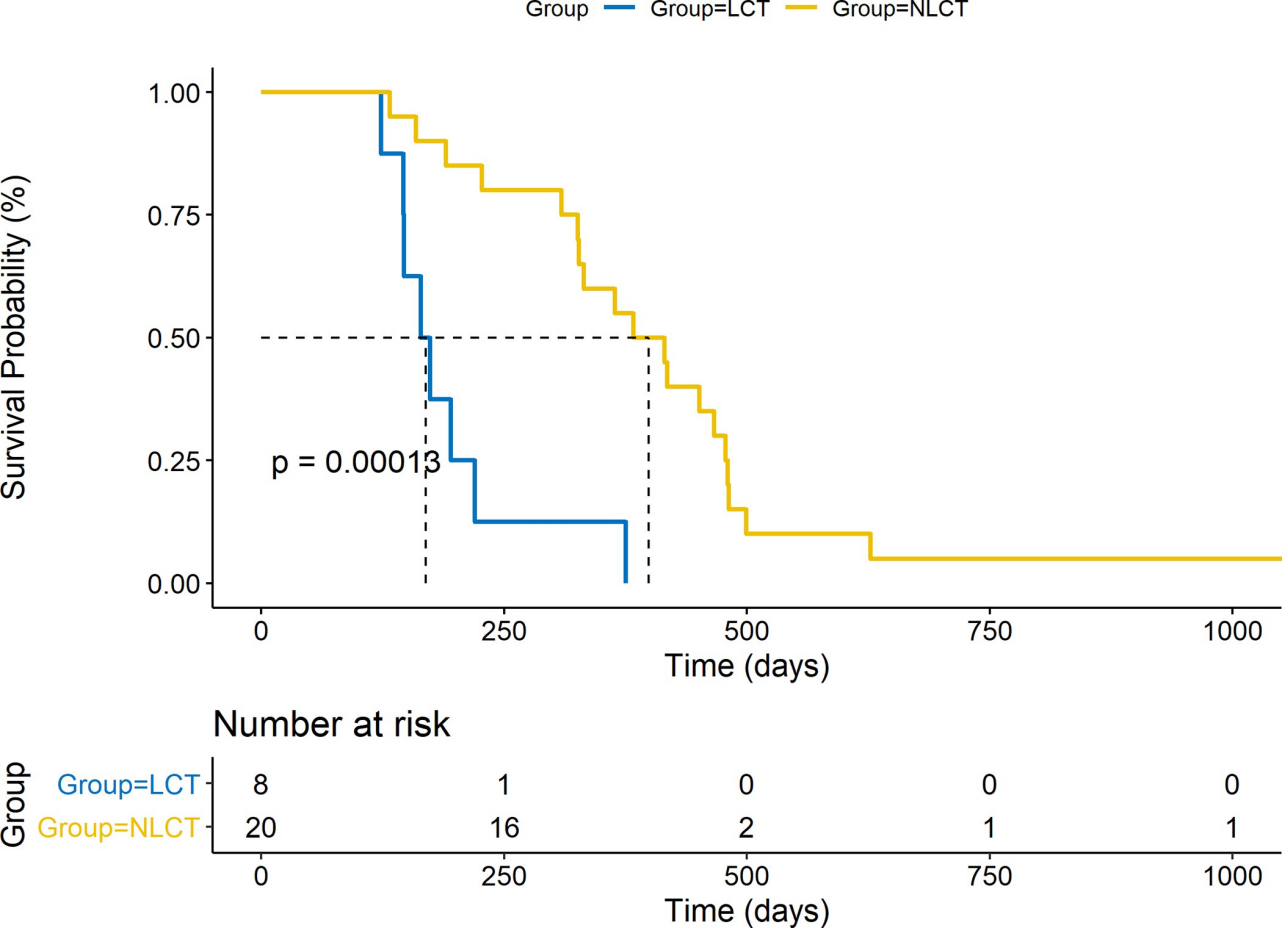
Average time-to-next-treatment (TTNT) analysis measured in days of large cell transformation (LCT) versus non-large cell transformation (NLCT) groups. The LCT group (blue line) demonstrated significantly decreased mean TTNT compared to the NLCT group (yellow line, HR 5.868, 95% CI 0.0623–0.4661, p = 0.0013).

Survival analysis was conducted by taking into account the time to death from diagnosis of SS (**OSF S1 Fig**) to LCT, comparing patients that underwent LCT to those that did not. There was no significant difference in OS between groups when considering time to diagnosis from SS to LCT (HR 1.297, 95% CI 0.491–3.424, p = 0.600). Upon analysis of CD8+ T cell counts relative to the median expression of all samples (“High” or “Low”), there were no differences in OS from SS diagnosis to death based on relative absolute CD8+ T cell count at diagnosis of SS (**OSF S2 Fig**, HR 1.726, 95% CI 0.6275–4.748, p = 0.29) or at most recent blood draw (**OSF S2B Fig**, HR 0.9202, 95% CI 0.3359–2.521, p = 0.872). When considering TTNT, defined as mean time to next systemic therapy, there was a significant difference observed between the two groups, with the LCT group demonstrating a significantly shorter TTNT than the NLCT group (**Fig 2**, HR 5.868, 95% CI 0.0623–0.4661, p = 0.0013), as would be expected in more aggressive disease phenotypes.

## Discussion

There are few studies which have explored the prognostic value of selected clinicopathologic factors to predict which patients with SS will eventually undergo LCT, although notably, LCT has been confirmed as being independently correlated with poor prognosis in multiple previous studies of cutaneous T cell lymphomas, including SS [5,8,10–16]. Studies investigating the clinical outcomes of LCT have determined that advanced stage at the time of LCT diagnosis is associated with poor prognosis [5,8]. Additionally, LCT at initial diagnosis of SS has been shown to be correlated with decreased survival [5,8,11]. Our study, however, demonstrated no difference in overall survival between LCT and NLCT groups, possibly secondary to small sample size, patient comorbidities, variability in treatments offered over the study’s nearly 10-year span, or socioeconomic factors that could not be controlled for. Elevated LDH has also been recognized as another independent poor prognostic marker for survival in patients with advanced-stage SS and LCT [5,17]. Furthermore, Novelli et al. found in a retrospective analysis of 134 cases that a TBSA> 50% involvement by either MF or SS was significantly correlated with decreased survival [18].

Our study suggests that there are clinical and pathologic factors that may predict the development of LCT. Increased extent of skin lesions (increased TBSA involvement) has previously been surmised to predict reduced disease-specific survival (DSS) and OS in patients with transformed MF, but these have not been studied specifically in SS [19]. Our study demonstrates that TBSA is predictive of LCT in SS. Because TBSA is seen as a marker of disease progression, TBSA may be a promising indicator of aggressive disease, rather than only a modifiable disease factor that is correlated with LCT. The same may be said for the presence of ulceration on pathologic specimens, as it correlated with increased TBSA involvement in our dataset.

It is well-accepted that CD8+ T cells play an important role in mediating anti-tumor immunity by eliminating tumor cells through recognition of tumor-associated antigens. In cancers such as colorectal, lung, breast, melanoma and glioblastoma, the infiltration of T cells, and particularly CD8+ T cells, is correlated with better prognosis [20,21]. There are promising immunotherapies in development and trials for various cancers that aim to boost CD8+ T cell-mediated anti-tumor immunity, which include adaptive cell transfer of tumor-reactive T cells (either native or engineered to express tumor specific T cell receptors or chimeric antigen receptors), dendritic cell cancer vaccines (DCVax), and immune checkpoint blockades (including anti-PD-1, anti-PD-L1, and anti-CTLA-4 antibodies) [22,23]. Our analysis poses an interesting question concerning the role of these non-neoplastic cells in SS. Previously, it has been demonstrated that a higher CD8+ T cell infiltrate in the skin is correlated with better prognosis in patients across plaque-stage MF, tumor stage MF, and CD30- PCTCL, and is correlated with less aggressive disease [24]. These results are supported by Hoppe et al. who describe a relationship between higher CD8+ T cell proportions and better survival in MF [25]. To the authors’ knowledge, this relationship is yet to be studied in SS. Our study demonstrated the stark contrast in absolute CD8+ T cell number in the peripheral blood between patients in the LCT and NLCT groups, with significantly decreased absolute counts in patients who underwent transformation, both before and after diagnosis of LCT. This observation may indicate that decreased peripheral CD8+ T cell count may also allow for persistence or continued resistance against LCT in SS. This is consistent with the demonstrated role of CD8+ T cells as anti-tumoral effector cells. Given the retrospective nature of the study, we were unable to obtain sequencing data for the markers of T cell activation that may have offered a more robust analysis of the functional capabilities of the T cells present in the peripheral blood. Further *in vitro* and *in vivo* studies are needed to understand these results in a mechanistic context.

Our study took advantage of the electronic health records to identify patients to be included in the analysis, however, this is a retrospective analysis in a disease with low prevalence, which may explain some of the unusually high hazard ratios seen in our results, and additionally may affect the interpretation of results. Larger studies may reveal patterns that we were not able to appreciate with a small number of patients. Given the low incidence of LCT, addressing this limitation may be difficult to overcome. Thus, future studies utilizing *in vitro* and *in vivo* laboratory experimentation may be required confirm the validity of our hypothesized mechanisms.

In conclusion, our 28-patient study identified the risk factors for LCT in patients diagnosed with SS. Here, we identify clinical and pathologic factors that may confer prognostic value for the incidence of LCT and assess their impact on OS. These include maximum TBSA %, peak LDH, presence of ulceration, and decreased levels of CD8+ cells in the peripheral blood. Although some of these factors may be clinical surrogates for disease progression, the significance of absolute CD8+ cell counts in the peripheral blood provides new insight into LCT risk in SS and implicates potentially novel therapeutic approaches in management. Greater absolute CD8+ cell count in the peripheral blood may serve as a protective factor against the development of LCT in SS. Furthermore, lesser numbers of CD8+ T cells may allow for persistence of the disease. Clinicians may use this insight in stratifying patient risk and deciding whether to escalate therapy, in conjunction with the other variables found to be significant in the study, such as maximum TBSA involvement, LDH level, or ulceration on biopsy specimen. Therapy targeted at increasing the number of CD8+ T cells in the peripheral circulation may serve to protect patients from developing transformation of their SS. These results indicate the need for further study into the mechanisms of tumor progression based on immune cell populations in patients with SS and need for a larger multi-center trial to create robust prognostic indices.

## Supporting information

S1 Fig Fig 1 demonstrates the Kaplan-Meier curves displaying differences in time from SS dx to death for patients who underwent large cell transformation (LCT) and patients who did not undergo large cell transformation (NLCT) (HR 1.297, 95% CI 0.491–3.424, p = 0.600). (TIFF)

S2 Fig Panel (A) demonstrates a Kaplan-Meier curve for overall survival from SS diagnosis to death, based on absolute CD8+ T cell count at dx of SS (HR 1.726, 95% CI 0.6275–4.748, p = 0.29). Panel (B) demonstrates a Kaplan-Meier curve for overall survival from SS diagnosis to death, based on absolute CD8+ T cell count at most recent blood draw (HR 0.9202, 95% CI 0.3359–2.521, p = 0.872). All hazard ratios are based on comparison of absolute CD8+ T cell Low (L) vs. High (H).
(TIF)

S1 File Supplement (https://osf.io/re2hd/?view_only=af032f917789428c9fc705d46ce44e3c): Data are available in the linked publicly available repository. (DOCX)

## Abbreviations

LCT: Large Cell Transformation
NLCT: Non-Large Cell Transformation
SS: Sezary Syndrome
Abs: Absolute
TBSA: Total Body Surface Area
LDH: Lactate Dehydrogenase
Dx: diagnosis
MF: Mycosis Fungoides
TTNT: Time to Next Treatment
OS: Overall Survival
HR: Hazard Ratio
CI: Confidence Interval
ISCL: International Society for Cutaneous Lymphomas
PET: Positron Emission Tomography
