# Supplementary figures and images for: Predictors of large cell transformation in patients with Sezary Syndrome—A retrospective analysis
Source: PLoS One. 2022 Nov 16;17(11):e0277655. doi: 10.1371/journal.pone.0277655 (PMC9668185; doi:10.1371/journal.pone.0277655)

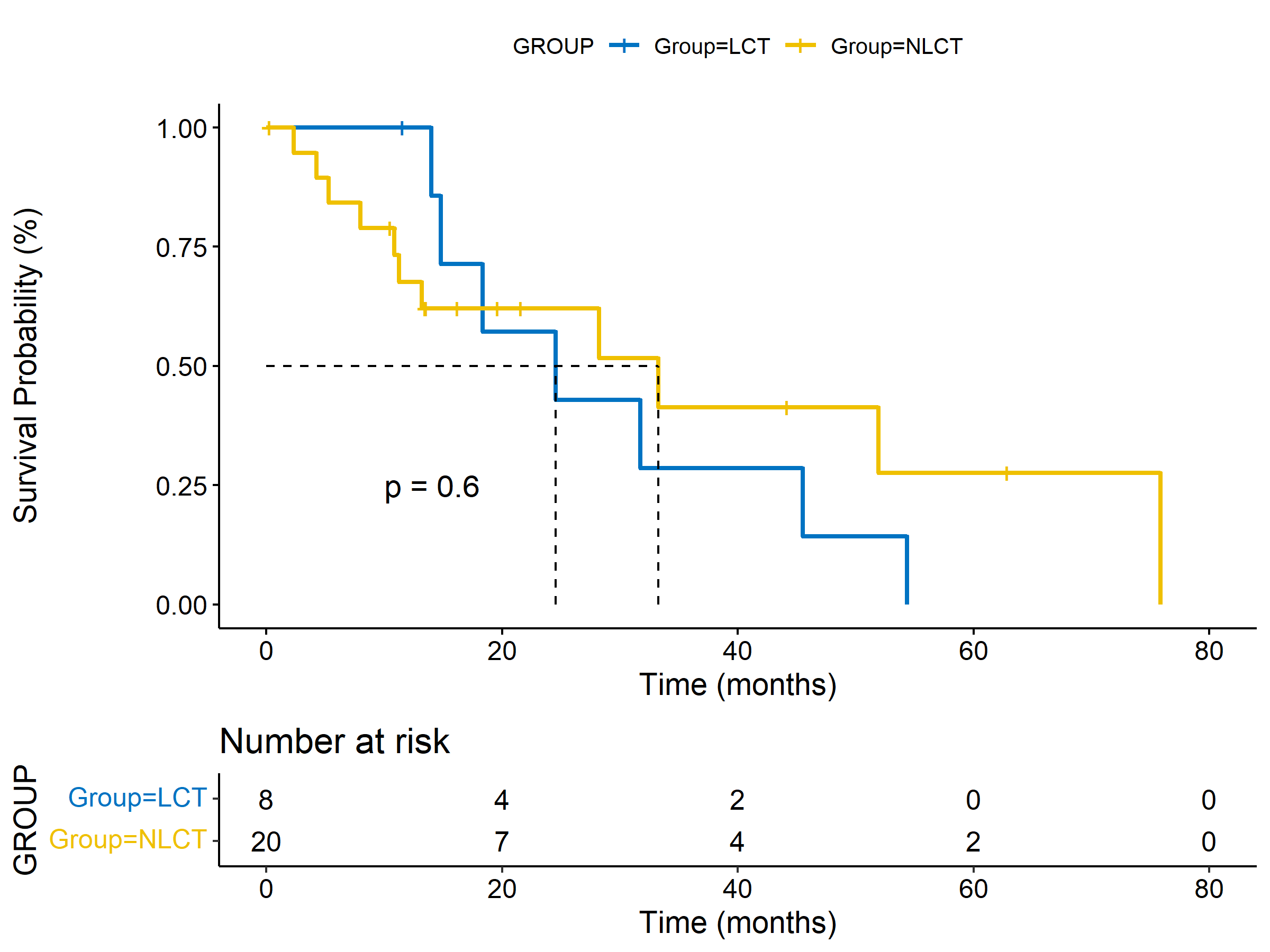

Supplement: S1 Fig — (TIFF) [file pone.0277655.s001.tiff]

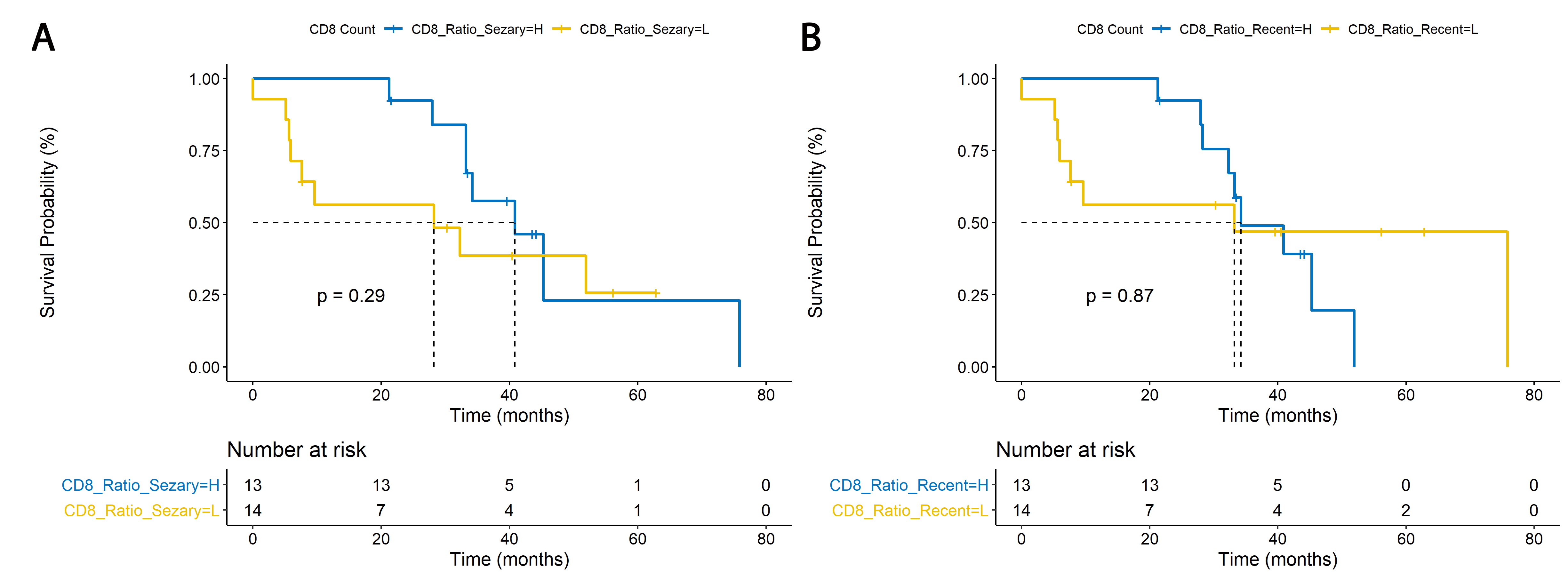

Supplement: S2 Fig — Panel (A) demonstrates a Kaplan-Meier curve for overall survival from SS diagnosis to death, based on absolute CD8+ T cell count at dx of SS (HR 1.726, 95% CI 0.6275–4.748, p = 0.29). Panel (B) demonstrates a Kaplan-Meier curve for overall survival from SS diagnosis to death, based on absolute CD8+ T cell count at most recent blood draw (HR 0.9202, 95% CI 0.3359–2.521, p = 0.872). All hazard ratios are based on comparison of absolute CD8+ T cell Low (L) vs. High (H). (TIF) [file pone.0277655.s002.tif]
