# Supplementary material for: Predictors of large cell transformation in patients with Sezary Syndrome—A retrospective analysis
Source: PLoS One. 2022 Nov 16;17(11):e0277655. doi: 10.1371/journal.pone.0277655 (PMC9668185; doi:10.1371/journal.pone.0277655)
Supplement: S1 File — (DOCX) [file pone.0277655.s003.docx]

**Supplement (**https://osf.io/re2hd/?view_only=af032f917789428c9fc705d46ce44e3c**):** Data are available in the linked publicly available repository.
